# Dynamic Thermal Response of Multiple Interface Cracks between a Half-Plane and a Coating Layer under General Transient Temperature Loading

**DOI:** 10.3390/ma17112478

**Published:** 2024-05-21

**Authors:** Mahsa Nourazar, Weilin Yang, Zengtao Chen

**Affiliations:** Department of Mechanical Engineering, University of Alberta, Edmonton, AB T6G 1H9, Canada; nourazar@ualberta.ca (M.N.); weilin4@ualberta.ca (W.Y.)

**Keywords:** multiple-crack problems, interface cracks, non-Fourier heat conduction, dislocation technique, thermal loading, singular integral equations

## Abstract

This paper explores the thermal behavior of multiple interface cracks situated between a half-plane and a thermal coating layer when subjected to transient thermal loading. The temperature distribution is analyzed using the hyperbolic heat conduction theory. In this model, cracks are represented as arrays of thermal dislocations, with densities calculated via Fourier and Laplace transformations. The methodology involves determining the temperature gradient within the uncracked region, and these calculations contribute to formulating a singular integral equation specific to the crack problem. This equation is subsequently utilized to ascertain the dislocation densities at the crack surface, which facilitates the estimation of temperature gradient intensity factors for the interface cracks experiencing transient thermal loading. This paper further explores how the relaxation time, loading parameters, and crack dimensions impact the temperature gradient intensity factors. The results can be used in fracture analysis of structures operating at high temperatures and can also assist in the selection and design of coating materials for specific applications, to minimize the damage caused by temperature loading.

## 1. Introduction

The behavior of heat flux near cracks can provide insights into stress concentrations and potential points of failure. In materials subject to thermal loading, such as metals in high-temperature environments, knowing how the heat temperature gradient is distributed helps predict where cracks are likely to grow or how they might propagate. While the traditional Fourier heat conduction model is generally effective for many engineering uses, it falls short when dealing with very small sizes and brief time periods, especially in smaller systems. Unlike the classical Fourier approach, non-Fourier heat conduction theory accounts for the finite speed of heat propagation, offering a more accurate depiction in scenarios involving rapid thermal transitions. This model has been particularly useful for researchers studying how heat affects cracks in materials [1,2,3,4,5,6]. Many researchers have studied the behavior of heat flux and the temperature gradient at crack tips. Chen and Hu [7] studied hyperbolic heat conduction within a cracked thermoelastic half-plane bonded to a coating, unveiling the significant impact of non-Fourier heat conduction on temperature distributions around the crack. Similarly, Wang and Han [8] investigated a finite medium with a crack under transient non-Fourier heat conduction, demonstrating the critical role of non-Fourier behavior in altering the thermal flux intensity factor and temperature distribution near the crack, challenging the conventional Fourier conduction paradigm. Hu and Chen [9] employed the dual-phase-lag theory to analyze transient heat conduction in a cracked half-plane, highlighting the theory’s enhanced capability to accurately capture the thermal behaviors around the crack compared to classical models. Fu et al. [10] focused on non-Fourier heat conduction in a functionally graded cylinder containing a cylindrical crack, revealing that non-Fourier effects have a significant influence on heat flux and temperature fields, especially in materials with graded properties. Wen et al. [11] presented a peridynamic model for non-Fourier heat transfer in orthotropic plates with uninsulated cracks. This innovative approach effectively captures complex heat transfer behaviors around cracks, offering valuable insights for predictive maintenance and the design of resilient materials.

In many other studies the heat conduction is used then to ascertain the thermal stresses in cracked materials [12,13,14,15,16]. In recent years, Wang and Schiavone [17] determined the temperature and thermal stresses of a finite Griffith crack perpendicular to the surface of an isotropic half-plane under uniform remote heat flux. Yang et al. [18] examined the dynamic behavior of a piezoelectric material strip containing a parallel crack exposed to thermal shock and transient electric loading with the use of non-Fourier heat conduction theory. Yang et al. [19] studied the interaction of two colinear cracks in an FGM layer subjected to sudden thermal shocks. They considered the non-Fourier effect using the dual-phase-lag heat conduction theory.

Thermal analysis of interface cracks has also been carried out by many researchers [20,21,22]. Ding, Zhou, and Li [23] investigated the behavior of interface cracks in a layered orthotropic strip subjected to thermal and mechanical loads and demonstrated the impact of material nonhomogeneity on thermal stress intensity factors. Zhang, Chen, and Li [24] then expanded our understanding by exploring non-Fourier heat conduction in materials with interface cracks, bridging classical and modern heat conduction theories. Kalinović et al. [25] focused on the thermal fracture characteristics, specifically the energy release rate and thermal stress intensity factor, of an interface crack in dissimilar elastic materials under temperature changes. Hu et al. [26] analyzed the interfacial crack initiation mechanism of thermal barrier coatings in isothermal oxidation processes. Lastly, Yang et al. [27] conducted a thermal and fracture analysis of colinear interface cracks in graded coating systems under ramp-type heating.

In this study, we performed a thermal analysis of a coated half-plane with multiple interface cracks under transient temperature loading. To do so, we utilized the distributed dislocation method, and the intensity factors of the temperature gradient were determined using non-Fourier, hyperbolic heat conduction. The paper concludes with numerical examples illustrating the results for the scenarios of one, two, and three interface cracks.

## 2. Formulation of the Problem

**Figure 1:**
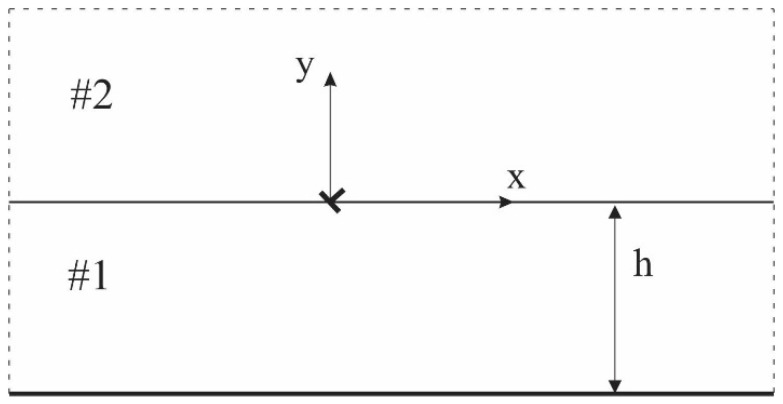
Schematic view of a single dislocation at an interface.

In this paper, we examine the interface of two materials, as shown in Figure 1. We can write the hyperbolic, non-Fourier heat conduction equation as follows [6] for finding the temperature distribution:

(1) $$k(\frac{\partial^{2}T}{\partial x^{2}}+\frac{\partial^{2}T}{\partial y^{2}})=\rho c\tau_{q}\frac{\partial^{2}T}{\partial t^{2}}+\rho c\frac{\partial T}{\partial t}$$

in which *k* is thermal conductivity, ρ is mass density, *c* is specific heat, and $\tau_{q}$ is thermal relaxation time. For initial conditions $T(x,y,0)=0,\;\dot{T}(x,y,0)=0$, after using Fourier and Laplace transformations, the temperature field can be written as follows:

(2) $$\left\{\begin{array}{cc} \overset{̑}{\bar{T_{1}}}\left(\omega ,y,s\right)=Ae^{\gamma_{1}y}+Be^{-\gamma_{1}y}\;\;\;\;\;\;\;\; & -h\leq y\leq 0\\ \overset{̑}{\bar{T_{2}}}\left(\omega ,y,s\right)=Ge^{-\gamma_{2}y} & 0\leq y \end{array}\right.$$

in which $\gamma_{i}=\sqrt{\left(\omega^{2}+\frac{\rho_{i}c_{i}\tau_{qi}}{k_{i}}s^{2}+\frac{\rho_{i}c_{i}}{k_{i}}s\right)}$ and $\overset{̑}{\bar{T_{1}}}\left(\omega ,y,s\right)=L\left\{F\left\{T\left(x,y,t\right)\right\}\right\};$ ω and *s* are Fourier and Laplace parameters; subscripts *i* = 1,2 refer to the variables of the coating and substrate materials, respectively; and *A*, *B*, and *G* are unknown functions to be determined. In the following formulations, the “¯” and “^” symbols are left out for simplicity.

### 2.1. Intact Half-Plane with a Thermal Coating under the Disturbance of a Single Thermal Dislocation

By introducing a temperature discontinuity $b_{T}(t)$ located at the origin, as shown in Figure 1, we can express the boundary conditions and continuity conditions for the temperature gradient at the location of the dislocation, as follows:

(3) $$\left\{\begin{array}{l} \;\;\;T_{2}(x,0^{+},t)-T_{1}(x,0^{-},t)=b_{T}(t)H(x)\\ \frac{\partial T_{1}(x,0^{-},t)}{\partial y}=\frac{\partial T_{2}(x,0^{+},t)}{\partial y}\\ \;\;\;T_{1}(x,-h,t)=0\\ \;\;\;T_{2}(x,\infty ,t)=0 \end{array}\right.$$

where $H\left(.\right)$ is the Heaviside step function. Applying Laplace and Fourier transformations to the above conditions, we arrive at the following:

(4) $$\left\{\begin{array}{l} \;\;\;T_{2}(\omega ,0^{+},s)-T_{1}(\omega ,0^{-},s)=b_{T}(s)[\frac{i}{\omega }+\pi \delta (\omega )]\\ \frac{\partial T_{1}(\omega ,0^{-},s)}{\partial y}=\frac{\partial T_{2}(\omega ,0^{+},s)}{\partial y}\\ \;\;\;T_{1}(\omega ,-h,s)=0\\ \;\;\;T_{2}(\omega ,\infty ,s)=0 \end{array}\right.$$

Through the above equations, the unknown functions in Equation (2), and accordingly, the temperature field, can be determined. The temperature gradient in the y-direction with a Cauchy singularity can be formulated as follows:

(5) $$\left\{\begin{array}{l} T_{1,y}=-\frac{b_{T}(s)}{2\pi }\frac{x}{x^{2}+y^{2}}+\frac{1}{\pi }\int_{0}^{\infty }\left[\frac{1}{\omega }\gamma_{1}A\left(e^{\gamma_{1}y}+e^{-{2\gamma }_{1}h}e^{-\gamma_{1}y}\right)+\frac{1}{2}e^{\left|\omega \right|y}\right]b_{T}(s)\sin\omega xd\omega \\ \;\;\;\;\;\;\;\;\;\;\;+\frac{1}{2}\gamma_{01}G_{0}e^{-\gamma_{01}y}b_{T}(s)\;\;\;\;\;\;\;\;\;\;\;\;\;\;\;\;\;\;\;\;\;\;\;\;\;\;\;\;\;\;\;\;\;\;\;\;\;\;\;\;\;\;\;\;\;\;\;\;\;\;\;\;\;\;\;\;\;\;\;\;\;\;\;\;\;\;\;\;-h<y\leq 0\\ T_{2,y}=-\frac{b_{T}(s)}{2\pi }\frac{x}{x^{2}+y^{2}}-\frac{1}{\pi }\int_{0}^{\infty }\left[\frac{1}{\omega }\gamma_{2}Ge^{-\gamma_{2}y}-\frac{1}{2}e^{-\left|\omega \right|y}\right]b_{T}(s)\sin\omega xd\omega \\ \;\;\;\;\;\;\;\;\;\;\;-\frac{1}{2}\gamma_{02}G_{0}e^{-\gamma_{02}y}b_{T}(s)\;\;\;\;\;\;\;\;\;\;\;\;\;\;\;\;\;\;\;\;\;\;\;\;\;\;\;\;\;\;\;\;\;\;\;\;\;\;\;\;\;\;\;\;\;\;\;\;\;\;\;\;\;\;\;\;\;\;\;\;\;\;\;\;\;\;\;\;\;\;\;\;\;\;\;\;\;\;\;0\leq y \end{array}\right.$$

in which $\gamma_{0i}=\sqrt{\left(\frac{\rho_{i}c_{i}\tau_{qi}}{k_{i}}s^{2}+\frac{\rho_{i}c_{i}}{k_{i}}s\right)}$, and functions *A* and *G* are given in the Appendix A.

### 2.2. Solution for the Intact Half-Plane with a Thermal Coating under General Thermal Loading

In this part of the study, our goal is to determine the temperature distribution and temperature gradient in the coating structure that is not affected by cracks, or in an intact half-plane with a coating. We begin by assuming that the temperature field in the undamaged medium is represented in the following form:

(6) $$\left\{\begin{array}{cc} T_{1}(\omega ,y,s)=Ce^{\gamma_{1}y}+De^{-\gamma_{1}y}\;\;\;\;\;\; & -h<y\leq 0\\ T_{2}(\omega ,y,s)=Ee^{-\gamma_{2}y} & h\leq y \end{array}\right.$$

We consider that the configuration is subjected to a sudden change in general temperature loading at the boundary *y* = −*h*; thus, the initial and boundary conditions for Equation (6) can be stated as follows:

(7) $$\left\{\begin{array}{l} \;\;\;\;T\left(x,y,0\right)=0\\ \;\;\;\;T_{2}(x,0^{+},t)=T_{1}(x,0^{-},t)\\ \frac{\partial T_{2}(x,0^{+},t)}{\partial y}=\frac{\partial T_{1}(x,0^{-},t)}{\partial y}\\ \;\;\;\;T_{1}(x,-h,t)=T_{0}e^{-\eta \left|x\right|}H(t)\\ \;\;\;\;T_{2}(x,\infty ,t)=0 \end{array}\right.$$

Applying Laplace and Fourier transforms to above conditions, we arrive at the following:

(8) $$\left\{\begin{array}{l} \;\;\;\;T\left(\omega ,y,0\right)=0\\ \;\;\;\;T_{2}\left(\omega ,0^{+},s)=T_{1}(\omega ,0^{-},s\right)\\ \frac{\partial T_{2}(\omega ,0^{+},s)}{\partial y}=\frac{\partial T_{1}(\omega ,0^{-},s)\;}{\partial y}\\ \;\;\;\;T_{1}(\omega ,-h,s)=2\frac{T_{0}}{s}\frac{\eta }{\eta^{2}+\omega^{2}}\\ \;\;\;\;T_{2}(\omega ,\infty ,s)=0 \end{array}\right.$$

After solving the non-Fourier heat conduction equation along with Equation (8), the temperature distribution and temperature gradient under transient loading can be written as follows:

(9) $$\left\{\begin{array}{l} T_{1}(x,y,s)=\frac{1}{2\pi }\int_{-\infty }^{\infty }\left(Ce^{\gamma_{1}y}+De^{-\gamma_{1}y}\right)e^{-i\omega x}d\omega \\ T_{1,y}=\frac{1}{2\pi }\int_{-\infty }^{\infty }\gamma_{1}(Ce^{\gamma_{1}y}-De^{-\gamma_{1}y})e^{-i\omega x}d\omega \;\;\;\;\;\;\;\;\;\;\;\;\;\;\;\;\;\;\;\;\;\;\;-h<y\leq 0\\ T_{2}(x,y,s)=\frac{1}{2\pi }\int_{-\infty }^{\infty }\left(Ee^{-\gamma_{2}y}\right)e^{-i\omega x}d\omega \\ T_{2,y}=-\frac{1}{2\pi }\int_{-\infty }^{\infty }\gamma_{2}Ee^{-\gamma_{2}y}e^{-i\omega x}d\omega \;\;\;\;\;\;\;\;\;\;\;\;\;\;\;\;\;\;\;\;\;\;\;\;\;\;\;\;\;\;\;\;\;\;\;\;\;\;\;\;\;\;\;\;\;\;\;\;0\leq y \end{array}\right.$$

Functions *C*, *D*, and *E* are given in the Appendix A.

### 2.3. Integral Equations for the Interface Crack Problem of the Half-Plane with a Thermal Coating

The dislocation method can be used to study how the multiple interface cracks react to the transient thermal loading. Here, the insulated interface cracks of a total number of N are represented by the distribution of thermal dislocations on their surfaces. The integral equation for the *j*-th crack can be formulated based on Equations (5) and (9) as follows:

(10) $$T_{,y}\left(x_{j}\left(p\right),y_{j}\left(p\right),s\right)=\sum_{k=1}^{N}L_{k}\int_{-1}^{1}K_{Qjk}\left(p,q,s\right)B_{Tk}\left(q,s\right)dq,\;\;\;\;\;\;-1\leq q\leq 1.$$

in which $x_{j}$(*p*) $=x_{cj}+L_{j}p,\;y_{j}(p)=0$; and *p* and *q* represent discretization points on the *j*-th and *k*-th cracks, respectively; $L_{j}$ is the half-length; and $\left(x_{cj},0\right)$ specifies the center point of the *j*-th crack.

To tackle the complex integral equations presented by Equation (10), we apply a collocation technique based on Chebyshev polynomials, a method refined by Erdogan et al. [28]. The negative values on the left side of Equation (10) correspond to the temperature gradient measured at the surface of the crack, assuming the surrounding medium is intact. When considering the kernel expressions of the integral equations, especially when *j* equals *k* and as *p* approaches *q*, we encounter a Cauchy-type singularity, which can be characterized in a specific mathematical form that will be detailed:

(11) $$K_{Qjj}(p,q,s)=\frac{a_{Tj,-1}\left(s,p\right)}{p-q}+\sum_{m=0}^{\infty }a_{Tj,m}\left(p\right)(p-q{)}^{m},\;\;\;\;\;\;\;\;\;\;\;j\in \{1,2,\ldots ,N\}.$$

To analyze the embedded cracks and determining thermal dislocation densities, integral Equation (10) should be solved simultaneously with the following crack closure conditions:

(12) $$\int_{-1}^{1}B_{Tj}(s,q)L_{j}dq=0\;\;\;\;\;\;\;\;\;\;\;\;\;\;-1\leq q\leq 1$$

The integral equations feature a Cauchy kernel, leading to a solution that can be expressed as follows:

(13) $$B_{Tj}\left(s,q\right)=\frac{g_{Tj}\left(s,q\right)}{\sqrt{1-q^{2}}}\;\;\;\;\;\;\;\;\;\;\;\;\;\;-1\leq q\leq 1.$$

The temperature gradient intensity factors at the tips of the *j*-th crack may be defined as follows:

(14) $$\left\{\begin{array}{l} K_{TRj}\left(s\right)=\underset{r\to 0}{\lim}\sqrt{2r}T_{j,y}\left(s,1\right),\\ K_{TLj}(s)=\underset{r\to 0}{\lim}\sqrt{2r}T_{j,y}\left(s,-1\right), \end{array}\right.$$

where *R* and *L* refer to the right and left crack tips, respectively, and r represents the distance from the crack tips along the crack line. When applying Equations (10)–(14) and making certain simplifications, the temperature gradient intensity factors for the crack become the following:

(15) $$\left\{\begin{array}{l} K_{TLj}(s)=-\pi \sqrt{L_{j}}[a_{Tj,-1}(s,-1)g_{Tj}(s,-1)]\\ K_{TRj}(s)=\pi \sqrt{L_{j}}[a_{Tj,-1}(s,+1)g_{Tj}(s,+1)] \end{array}\right.$$

Numerical inversion of the Laplace transform is then performed using Stehfest’s method [29] as follows:

(16) $$\left\{\begin{array}{l} \;\;\;\;\;\;\;K_{Tk}(t)=\frac{\ln2}{t}\sum_{m=1}^{M}H_{m}K_{Tk}\left(\frac{\ln2}{t}m\right),\;\;\;\;\;\;\;\;\;\;\;\;\;\;\;\;\;\;\;\;\;\;\;\;\;\;\;\;\;\;\;\;k=R,L\\ H_{m}=(-1{)}^{\frac{M}{2}+m}\sum_{n=\left[0.5\left(m+1\right)\right]}^{\min(\frac{M}{2},m)}\frac{n^{\frac{M}{2}}\left(2n\right)!}{(\frac{M}{2}-n)!n!(n-1)!(m-n)!(2n-m)!} \end{array}\right.$$

in which *M* = 8 according to [29].

## 3. Results and Discussion

In this section, we provide numerical illustrations of the results. The temperature gradient intensity factor is computed temporally for configurations with both a single crack and multiple cracks. The materials are chosen as in [7], where $\frac{k_{1}}{c_{1}\rho_{1}}=2\frac{k_{2}}{c_{2}\rho_{2}},$ $k_{1}=0.5k_{2}$, $\tau_{1}=0.4,$ and $\tau_{2}=1,$ and the thickness of the coating layer is considered *h* = 2*L*, except where noted otherwise. To normalize the outcomes, we define $K_{0}=\left|T_{0}\right|/\sqrt{L}$, $t_{0}=L^{2}c_{1}\rho_{1}/k_{1}$.

### 3.1. Finite Element Verification

**Figure 2:**
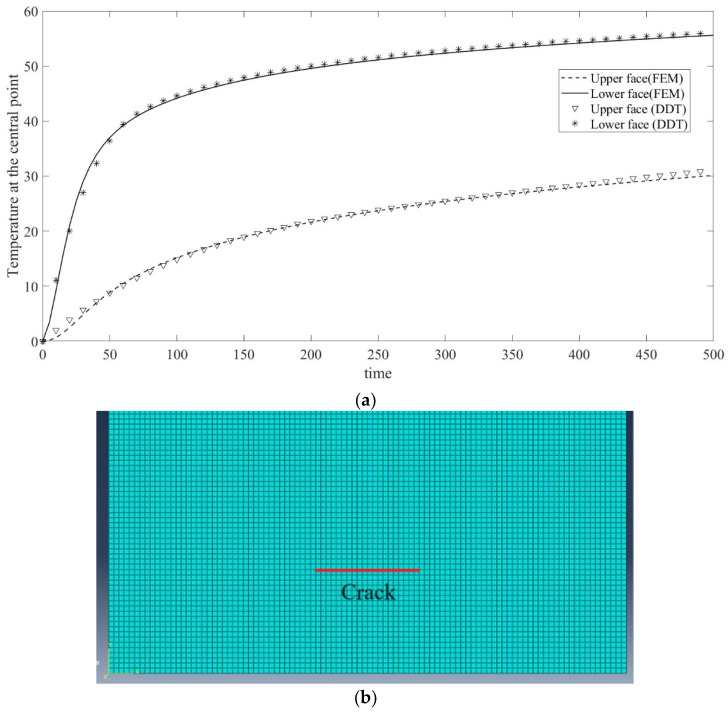
(**a**) Temperature variation at the central point on the upper and lower crack faces. (**b**) Finite element model of the crack problem simulated in Abaqus.

In order to verify our theoretical model, a comparison of the results of a finite element model (FEM) and the present distributed dislocation technique (DDT) is shown in Figure 2a. Specifically, the temperature at the central point of the upper and lower crack faces is shown based on the Fourier heat conduction. In the comparison, a simple homogeneous steel material is considered with $\rho =7870\frac{kg}{m^{3}},\;k=20\frac{W}{mK},\;C=450\frac{J}{kgK}$. The finite element model is built in Abaqus, as shown in Figure 2b. After a mesh sensitivity study of the convergence of the model, the quad element size is set as 1 mm. We set the height and width of the strip as 10 cm, large enough in comparison with the length of the crack of 1 cm to simulate the corresponding half-plane crack problem. A transient $T_{0}=100\;K$ is applied on the lower surface, and then the temperature of the central point of the crack is taken and compared with the results obtained through the DDT method. The results of the DDT method match the finite element result very well, which verifies the correctness of the present DDT method.

### 3.2. Single-Interface-Crack Problem

**Figure 3:**
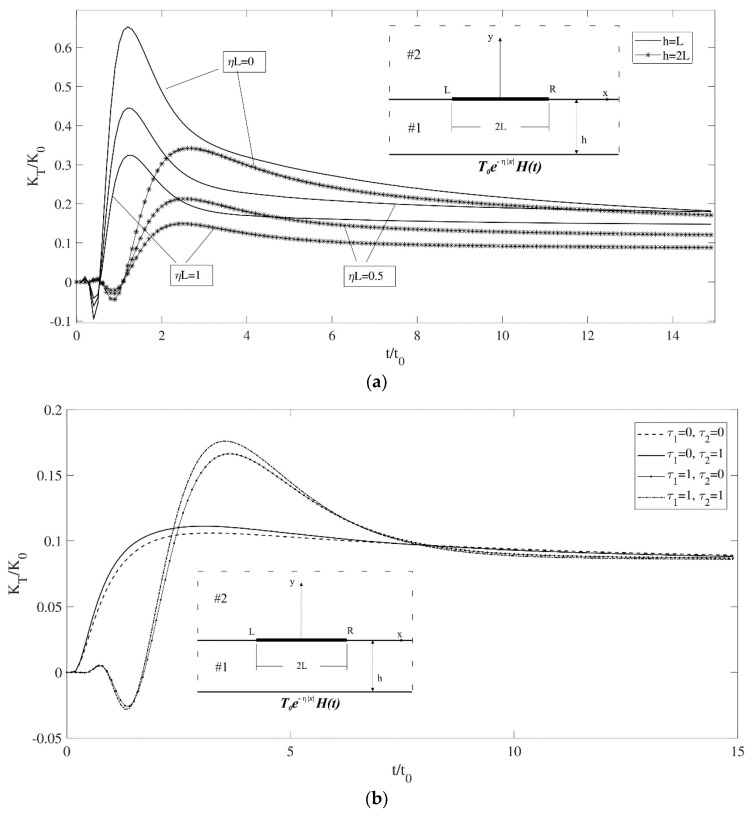

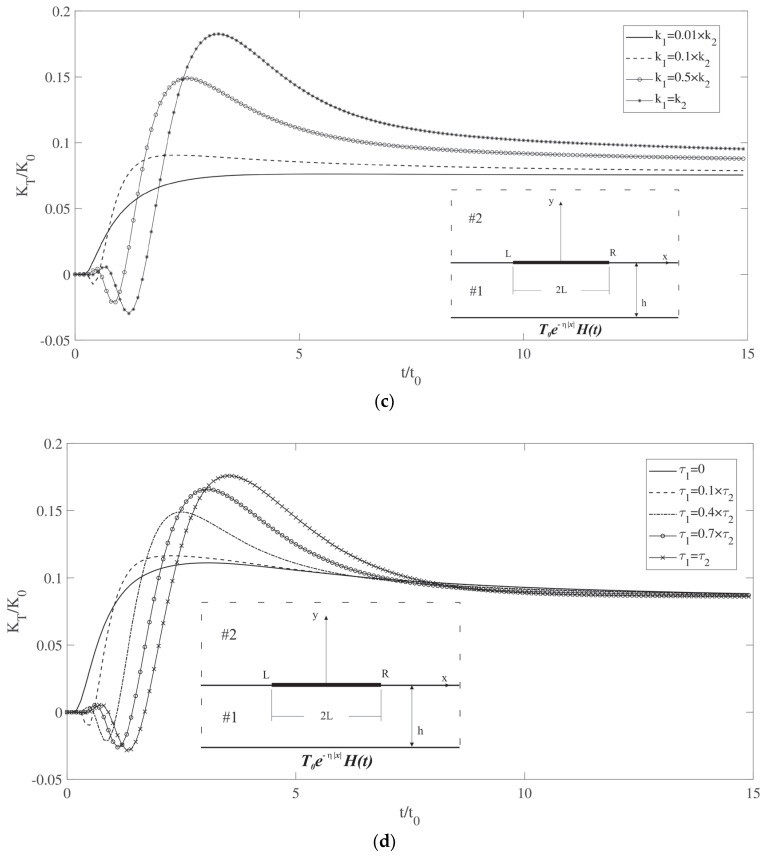
(**a**) Temperature gradient intensity factor for a single crack versus time for different values of η and *h*. (**b**) Temperature gradient intensity factor for a single crack versus time (symmetrical, *ηL* = 1). (**c**) Temperature gradient intensity factor for a single crack versus time for different values of the coating’s thermal conductivity (symmetrical, ηL = 1). (**d**) Temperature gradient intensity factor for a single crack versus time for different values of the coating’s relaxation time (symmetrical, ηL = 1).

In the first example, a single interface crack is considered. The variation in the temperature gradient intensity factor versus time for different values of coating thickness and loading parameter is depicted in Figure 3a. Decreasing the thickness leads to the peak value occurring sooner and more intensely, while increasing the η value results in a lower peak value and quicker stabilization.

Figure 3b shows the variation in temperature gradient intensity factors versus time for different values of $\tau_{q}$ for both materials. The thickness of the coating is set as *h* = 2*L*. The crack is located at the center, resulting in the right and left tips exhibiting identical intensity factors. When the relaxation time, $\tau_{q}$, is set to zero for both materials, parabolic heat conduction behavior is observed. If $\tau_{q}$ is adjusted to 0.5 for material #1 and remains at 0 for material #2, the graphic behavior shifts to hyperbolic heat conduction. This indicates that material #1 plays a dominant role in determining the influence of the relaxation time. Conversely, increasing the relaxation time for material #2 impacts the peak value, resulting in its elevation.

The variation in the temperature gradient intensity factor over time for an interface crack is illustrated in Figure 3c for different values of thermal conductivity of the coating material. This reveals that higher thermal conductivities lead to higher intensity factors and delayed stabilization. Furthermore, Figure 3d presents the influence of the relaxation time of the coating material, indicating that a greater relaxation time augments both the peak value and the time of its occurrence, though it bears no impact on the final, stabilized value.

### 3.3. Multiple-Interface-Crack Problem

**Figure 4:**
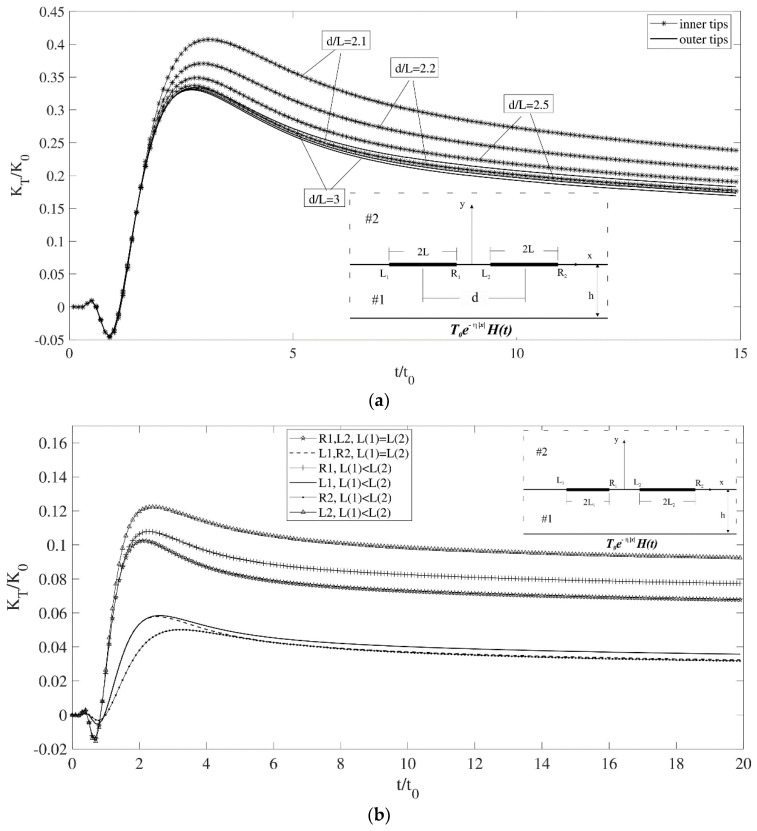
(**a**) Temperature gradient intensity factor for two identical cracks versus time (ηL = 0). (**b**) Temperature gradient intensity factor for two colinear cracks versus time (ηL(1) = 1).

In the following example, two identical interface cracks are considered, and the effect of crack spacing on the intensity factors is shown in Figure 4a. As expected, when decreasing the center-to-center distance between cracks, the increase in intensity factor for the inner tips is more significant than for the outer tips. In the next example, shown in Figure 4b, we examine the interaction between two cracks and assess how the lengths of the cracks affect the temperature gradient intensity factor. With cracks of equal length, the problem presents symmetry, resulting in identical values for the inner and outer tips. When the length of the second crack increases, its inner tip exhibits the highest peak value, while its outer tip attains the lowest peak value due to its increased distance from the peak heat point. Additionally, the temperature gradient intensity factors for both tips of the first crack increase.

**Figure 5:**
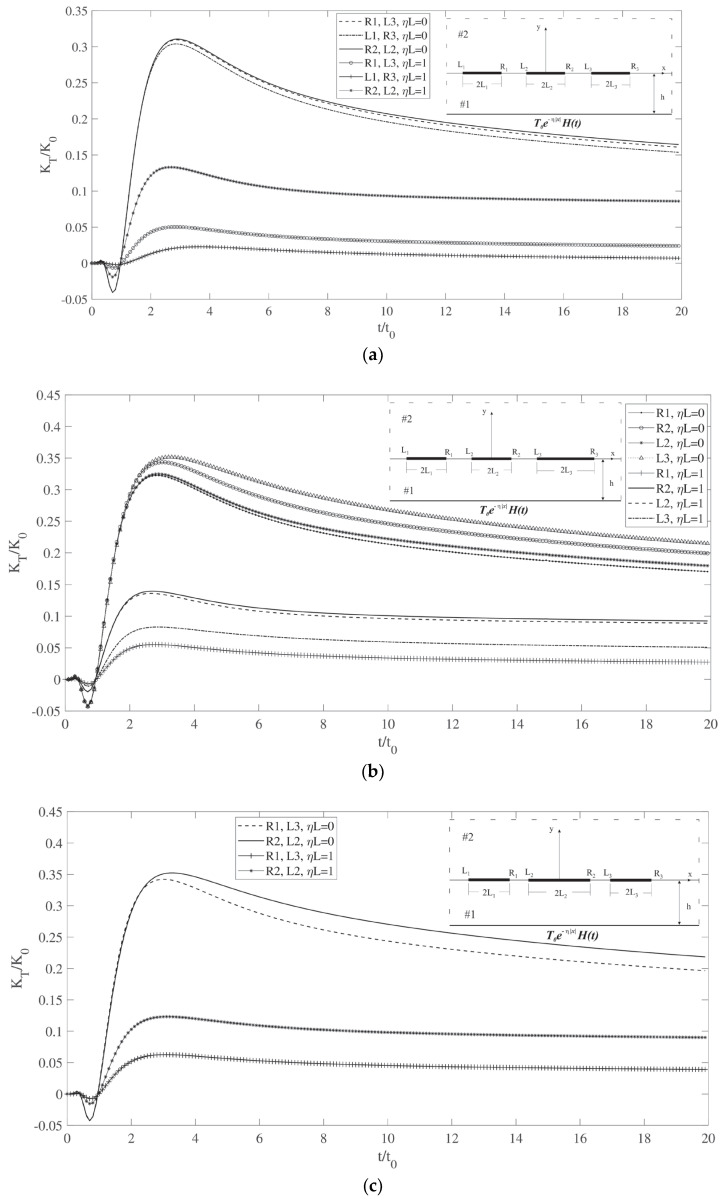
(**a**) Temperature gradient intensity factor for three colinear cracks with different lengths versus time (*L*(1) = *L*(2) = *L*(3) = *L*). (**b**) Temperature gradient intensity factor for three colinear cracks with different lengths versus time (*L*(1) = *L*(2) = *L* < *L*(3)). (**c**) Temperature gradient intensity factor for three colinear cracks with different lengths versus time (*L*(1) = *L*(3) = *L*, *L* < *L*(2)).

The final example illustrates the variation in the temperature gradient intensity factor over time for three colinear cracks of differing lengths under two loading conditions *ηL* = 0 and *ηL* = 1. Figure 5a shows the temperature gradient intensity factor for three colinear cracks with equal lengths *L*(1) = *L*(2) = *L*(3) = *L*. The symmetry across the cracks results in equal intensity factors for the corresponding tips. When ηL = 0, the middle crack exhibits the highest values, while the other tips show slightly lower values. As ηL increases to 1, the temperature’s descending trend significantly shifts the peak values, with the middle crack tips peaking first, followed by adjacent tips, and the lowest values at the outermost tips. In Figure 5b, with the right crack being longer, the highest intensity factor is seen when ηL = 0. The *L*_3_ tip exhibits the greatest value due to the crack’s length and interactions with the others. The intensity factor *R*_2_ is elevated compared to *L*_2_ and *R*_1_ because of these interactions. Under ηL = 1, temperature changes markedly affect the peak values, with the tips of the middle crack showing the highest intensity factors, and the right tip’s value is slightly higher due to its proximity to *L*_3_. Finally, Figure 5c depicts a scenario with the central crack being the longest, displaying symmetry and the highest intensity factors in both loading conditions.

## 4. Conclusions

This research presents the thermal behavior of multiple cracks situated at the interface of a coated half-plane when subjected to transient thermal loading. The temperature distribution was analyzed using the hyperbolic heat conduction theory and distributed dislocation technique (DDT). Numerical examples are presented to show the effect of the coating thickness, loading parameter, crack size and distribution, and thermal properties on the dynamic temperature gradient intensity factors. The numerical results demonstrate that when the coating is thicker, the temperature gradient intensity factor shows a lower and delayed peak value. On the other hand, the distribution of the transient thermal loading plays an important role in the thermal response of the coating system. In particular, by increasing the value of η, the peak value of the thermal intensity factor decreases and stabilizes to the steady-state value sooner. Crack length also plays a significant role: longer cracks lead to higher peaks and delayed stabilization of the transient thermal intensity factor, reflecting a direct correlation between crack size and transient thermal response. For given crack sizes, when the spacing between the cracks decreases, crack interactions are enhanced and the peak values of the thermal intensity factors increase. The thermal conductivity of the coating material plays a vital role in the thermal response of the cracked coating structure. When thermal conductivity of the coating material decreases, the peak value of the thermal intensity factor decreases accordingly, implying that low thermal conductivity is preferrable to avoid interface failure of the thermal coating. The thermal relaxation time of the coating material dominates the transient thermal response of the cracked coating structure. Increasing the relaxation time of the coating material increases the peak value of the thermal intensity factors and the time when it occurs. Further experimental study on non-Fourier heat conduction in coating structures with multiple interface cracks will be beneficial in promoting the application of the current work in thermal coating design.

## Data Availability

Data are contained within the article.

## Nomenclature

A,B,G	Unknown coefficients of temperature caused by one dislocation
$b_{T}(t)$	Temperature discontinuity
$B_{Tk}(t)$	Dislocation densities
*c*	Specific heat
C,D,E	Unknown coefficients of temperature in the intact medium
h	Vertical distance between the interface and the boundary
H(x)	Heaviside step function
*k*	Thermal conductivity
$K_{Qjk}\left(s,t\right)$	Kernels of integral equations
$K_{TL},K_{TR}$	Mode I stress intensity factor for the left and right crack tips
L	Half-lengths of the crack
N	Number of cracks
*p*,*q*	Collocation points of Chebyshev polynomials
s	Laplace transform parameter
*t*	Time
$T_{i,y}$	Temperature gradient in the y direction
$T_{i}$	Temperature
$\gamma_{i}$	Roots of the characteristic equation
δ(ω)	Dirac delta function
η	Loading parameter
ρ	Mass density
$\tau_{q}$	Relaxation time
ω	Fourier transform parameter

## Appendix A

The functions in Equation (5) are as follows:

(A1) $$G=\frac{\left[1+\frac{1}{2}\left(e^{-{2\gamma }_{1}h}-1\right)\right]}{\left[1+\frac{1}{2}\left(-\frac{\gamma_{2}}{\gamma_{1}}+1\right)\left(e^{-{2\gamma }_{1}h}-1\right)\right]}b_{T}(s)\left[\frac{i}{\omega }+\pi \delta \left(\omega \right)\right]$$

(A2) $$A=\frac{1}{2}\{\frac{\left[1+\frac{1}{2}\left(e^{-{2\gamma }_{1}h}-1\right)\right]}{\left[1+\frac{1}{2}\left(-\frac{\gamma_{2}}{\gamma_{1}}+1\right)\left(e^{-{2\gamma }_{1}h}-1\right)\right]}(-\frac{\gamma_{2}}{\gamma_{1}}+1)-1\}b_{T}(s)[\frac{i}{\omega }+\pi \delta (\omega )]$$

The functions in Equation (9) can be found as follows:

(A3) $$E=\frac{2\frac{T_{0}}{s}\frac{\eta }{\eta^{2}+\omega^{2}}e^{\gamma_{1}h}}{\left[1-\frac{\left(\gamma_{1}+\gamma_{2}\right)}{{2\gamma }_{1}}\left(1-e^{2\gamma_{1}h}\right)\right]}$$

(A4) $$C=\frac{\left(\gamma_{1}-\gamma_{2}\right)}{{2\gamma }_{1}}E$$

(A5) $$D=\frac{\left(\gamma_{1}+\gamma_{2}\right)}{{2\gamma }_{1}}E$$
